# Technetium-99m Tricarbonyl Labeled a Broad-spectrum Quinolone as a Specific Imaging Agent in Infection Diseases

**Published:** 2017

**Authors:** Seyed Javad Khoramrouz, Mostafa Erfani, Mitra Athari Allaf

**Affiliations:** a *Science and Research branch, Islamic Azad University, P.O. Box: 14515-775, Tehran, Iran.*; b *Radiation Application Research School, Nuclear Science and Technology Research Institute (NSTRI), P.O.Box: 14395-836, Tehran, Iran.*

**Keywords:** Infection, ^99m^Tc-carbonyl, Gemifloxacin, Quinolone, Imaging

## Abstract

Nuclear medicine imaging has been used to localize infection sites, and efforts have been continued to develop modified infection specific radiopharmaceuticals. In this study gemifloxacin as a broad-spectrum quinolone has been labeled with [^99m^Tc (CO)_3 _(H_2_O)_3_]^+ ^core in order to evaluate its feasibility as an infection imaging agent for *in-vivo* use. The stability of radioconjugate was checked in human serum at 37 °C and biodistribution was studied in mice. Labeling yield of > 95% was obtained corresponding to a specific activity of 0.14 GBq/μmol. The radioconjugate showed good stability in human serum. Our main achievement was the high accumulation in the infected muscle in mice (T/NT = 2.93 ± 0.3 at 1 h post injection), which may diagnostically be beneficial for differentiate sites of infection from sites of inflammation.

## Introduction

Despite the progress in treatment of infectious diseases, infections remain among the most common and costly causes of death and disease around the world, especially in developing countries. The early diagnosis of infection from sterile inflammation is one of the most common problems in nuclear medicine and for this reason several radiopharmaceuticals have been developed to resolve the solution for this difficult situation. However, there are many limitations in developing specific radiopharmaceuticals for clear distinction between infection and inflammation.

^67^Ga-Citrate, being the most basic radiotracer for this purpose, has a high sensitivity for acute and chronic infections and sterile inflammation (1). There are drawbacks ascribe to this radiopharmaceutical, including its long physical half-life and high-energy radiation. A number of other radiopharmaceuticals labeled with different radioisotopes have been already used, but an ideal agent has not yet been found. Polyclonal and monoclonal immunoglobulins (2, 3) and liposomes labeled with ^99m^Tc (4-6) are nonspecific tracers and may cause certain difficulties. Antibody fragments and antigranulocyte antibodies, chemotactic peptides, cytokines and interleukins also have certain limitations (7). Autologous leukocytes labeled with ^111^In are still considered by many to be the gold standard for localization of infections but the product must be prepared in a sterile environment. Although leukocytes labeled with ^99m^Tc have the advantages of a low radiation to the patient and an ideal energy, this agent is less stable than ^111^In-labeled leukocytes (8). Antimicrobial peptides, produced by phagocytes, epithelial cells, endothelial cells, and many other cell types, are an important component of natural immunity against infection (9). In several previous studies, the ^99m^Tc labeled cationic antimicrobial peptide derived from human ubiquicidine (UBI) was introduced for detection of bacterial and fungal infections (10-12). 

Broad spectrums of antibiotic agents have been suggested as promising diagnostic tools for the detection of infected lesions. The antibiotic molecules accumulate at the site of infection due to their metabolization by microorganisms (13). The majority of the various antibiotics studied in this regard are those of the quinolones family, second and third generation cephalosporins (14-17). Ciprofloxacin as a quinolone antibiotic labeled with ^99m^Tc has shown a high sensitivity and specificity for infection imaging (18). This antibiotic binds to the DNA gyrase enzyme of bacteria and has shown good accuracy in hip prosthesis infections (19). However, antibiotics encounter the problem of antibiotic resistant bacteria, which is attributed to ciprofloxacin as well (20-23). 

Gemifloxacin is an oral broad spectrum quinolone antibacterial agent used in the treatment of acute bacterial aggravation of chronic bronchitis and mild to moderate pneumonia. Gemifloxacin has been shown to be active against most strains of the aerobic gram positive, aerobic gram negative and some other microorganisms (24). In order to use the increased ability of the gemifloxacin for localization of infection in the present study, radiolabeling of the gemifloxacin with ^99m^Tc via carbonyl core was evaluated. Optimization of labeling condition, stability in human serum, lipophilicity, binding with Staphylococcus aureus and biodistribution in infected mice for the labeled compound were studied.

## Experimental

*General*

Gemifloxacin mesylate obtained from Life Sciences Pharmaceuticals (LSP). All other chemicals obtained from Sigma-Aldrich and were used without further purification. Technetium-99m as sodium pertechnetate (Na^99m^TcO_4_) was obtained from an in-house ^99^Mo/^99m^Tc generator using 0.9% saline. All reactions were performed and monitored using an analytical reverse-phase high performance liquid chromatography (RP-HPLC) on a JASCO 880-PU intelligent pump HPLC system (Tokyo, Japan) equipped with a multiwavelength detector and a flow-through Raytest-Gabi γ-detector. CC 250/4.6 Nucleosil 120-5 C-18 column from Teknokroma was used for HPLC. 0.1% trifluoroacetic acid/water (Solvent A) and 0.1% trifluoroacetic acid/acetonitrile (Solvent B) were used as a mobile phase in the following gradient: 0 min 95% A (5% B), 5 min 95% A (5% B), 25 min 0% A (100% B), 30 min 0% A (100% B), flow = 1 mL/min, λ = 280 nm. Radioactivity measurements were carried out using Na (Tl) scintillation counter (ORTEC Model 4001 M Minibin & Power Supply).

*Tricarbonyl*

The precursor [^99m^Tc (CO)_3_(H_2_O)_3_]^+^ was prepared according to the reported procedure by Mirshojaei et al [16]. Briefly, in a closed vial 4.5 mg Na_2_CO_3_, 5.5 mg NaBH_4_ and 20 mg sodium potassium tartarate were added and the vial was flushed with CO and after that the 1100 MBq activity that was eluted from a commercial ^99^Mo/^99m^Tc generator was added and heated to 95 °C for 30 min. After cooling the vial to room temperature, using 1 N HCl the solution was neutralized to pH = 7. 

*Radiochemical analysis*

Radiochemical purity of the precursor was checked by TLC (silica gel 60 Merck) using 99.9% methanol, 0.1% concentrated HCl as a solvent. The tricarbonyl precursors reveal a retention factor (Rf) of 0.3, and unreacted ^99m^TcO4^-^ show a Rf of 0.7 while Colloidal TcO_2_ remain at the origin. The radioactivity was quantified by cutting the strip (1.5 × 10 cm^2^) into 1 cm pieces and counting in a well type gamma counter. 

*Preparation of* ^99m^ *Tc(CO)*_3_ *-gemifloxacin*

A stock solution of gemifloxacin (concentration 20 mg/mL) was prepared by dissolving gemifloxacin in water; from this stock solution different amounts of gemifloxacin (0.5-5 mg) were carefully transferred to a vial. To this solution [^99m^Tc (CO)_3_(H2O)_3_]^+^ precursor (100 µL, 370 MBq) was added and while various range of pH (1-9) was adjusted, its labeling was completed by incubation of vial in a boiling water bath for 30 min. After cooling down to room temperature (15 min) the formulation was checked for preparation of complex. 

*In-vitro Stability*

Stability of ^99m^Tc(CO)_3_-gemifloxacin was evaluated in NaCl 0.9% (W/V) and in human serum. Aliquots were taken out at 1, 4, 6, 12 and 24 h post reconstitution at room temperature and analyzed by HPLC. A 100 μL of labeled formulation was added to 1 mL of freshly prepared human serum, and the mixture was incubated in a 37 °C environment. Also 100 μL aliquots were removed at the same time points and treated with 100 μL of alcohol. Samples were centrifuged for 5 min at 3000 rpm to precipitate serum proteins and for supernatants, chromatography was performed. 

*Log P values*

For partition coefficient, 0.5 mL of the ^99m^Tc labeled formulation was mixed with 0.5 mL of octanol in a 2 mL micro tube. The tube was vigorously vortexed over a period of 10 min and centrifuged at 3000 g for 5 min. Three aliquots of 100 μL were sampled from each layer and counted in the γ counter. The averaged activities from the aqueous and the octanol layers were used to calculate the log P values. The octanol-to-water partition coefficient (P_o/w_) was calculated by dividing the counts of the octanol phase by that of the aqueous phase.

*Binding to bacteria*

100 µL of the ^99m^Tc(CO)_3_-gemifloxacin was transferred to a test tube. Then, 0.9 mL of 50% (v/v) 0.01 M acetic acid in phosphate buffer (pH = 7.5) containing approximately 1×10^8 ^colony forming units (CFU) per mL viable *S. aureus* was added. The mixture was incubated for 1 h at 4 ºC and thereafter the vials were centrifuged in a pre-cooled centrifuge for 5 min at 2000 g at 4 ºC. The supernatant was removed, and analyzed by HPLC method for evaluation of stability of ^99m^Tc labeled compound in incubated medium and binding condition. The bacterial pellet was gently re-suspended in 1 mL of buffer and re-centrifuged as above. The supernatant was removed and the radioactivity in the bacterial pellet was measured by a gamma counter for percent of bounded activity.

*Biodistribution in infected mice*

Animal experiments were performed in compliance with the regulations of our institution and with generally accepted guidelines governing such work. Male Balb/c mice, weighing 25-30 g were infected by injection 0.1 mL of saline containing 1×10^8 ^CFU *S. aureus* bacteria into right tight muscle. After 24 h, a group of three balb/c mice received 3.7 MBq of ^99m^Tc(CO)_3_-gemifloxacin in volume of 0.1 ml via a tail vein. The mice were sacrificed at different post injection times (1, 4 and 24 h) and the tissues and organs of interest were collected, immediately weighed and counted in a NaI well-type γ-counter. The uptake was expressed as the percentage of the injected dose per gram tissue (%ID/g tissue). The values are expressed as mean ± SD. 

*Scintigraphy studies*

At different time point after injection, accumulation of the tracer in infected area was assessed by planar scintigraphy using the single head gamma camera (small area mobile, Siemens, 140 KeV high sensitivity parallel whole collimator and 10% window around 140 KeV). Before the imaging mice were anesthetized with 0.05 mL ketamine 10% (3.3 mg) and 0.05 mL xylazine 2% (1.33 mg) intra-peritoneally. After about 5 min, the animal was fixed on a board by covering with pieces of cloth for immobilization during the scanning.

## Results and Discussion

**Figure 1.**
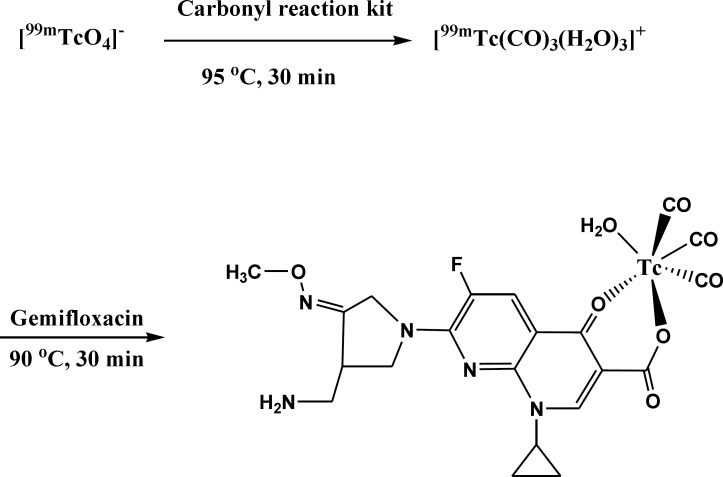
preparation and proposed structure of labeled gemifloxacin via carbonyl core

The significant property of the precursor [^99m^Tc (CO)_3_(H_2_O)_3_]^+^ provided by Alberto *et al*. (25) leads to further interest to the development of new ^99m^Tc-based radioconjugates. The technetium tricarbonyl core is able to forms highly stable complexes with a wide range of ligand systems containing donor atom such as nitrogen, oxygen and sulfur (26, 27). Previous study in structural characterization of rhenium(I) tricarbonyl complexes with the bidentate ligands showed that bidentate ligands react readily with the precursor to give complexes of the type [Re(CO)3(LL)X] (where LL = bidentate ligand) with square bipyramidal geometry which may be further elaborated for potential radiopharmaceutical applications (28). Due to presence of electron donor atoms such as oxygen in gemifloxacin structure, the tricarbonyl precursor can easily react with it and with replacement of its water component a complex is formed (Figure 1). Although the exact complex structure is not known, the proposed structure of the bidentate radiocomplex will have square bipyramidal geometry (28). Further researches seem to be required to determine the exact structure of this new conjugate.

**Figure 2:**
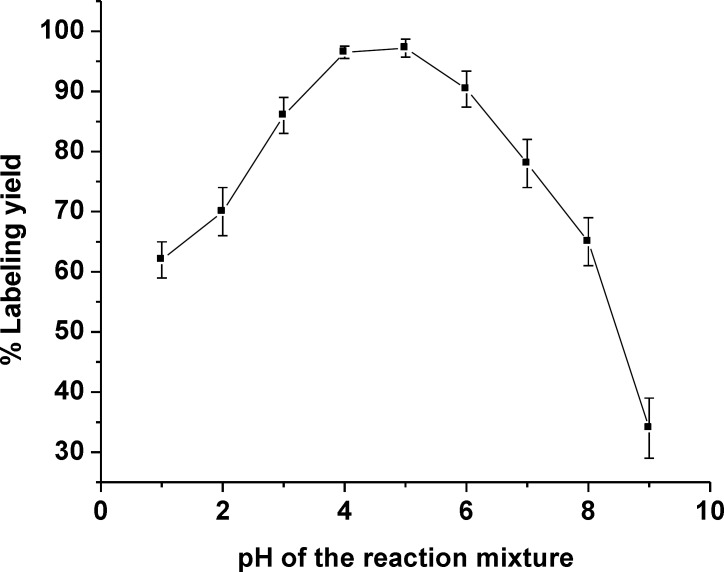
The effect of pH on the labeling yield of ^99m^Tc(CO)_3-_Gemifloxacin

**Figure 3:**
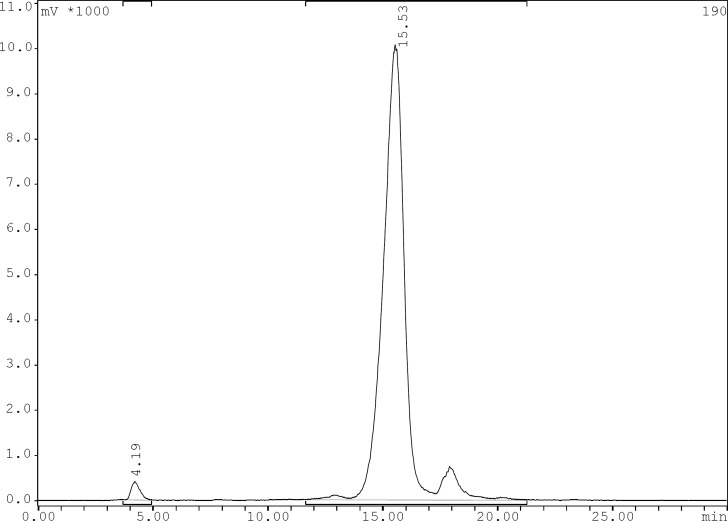
Reversed phase HPLC chromatogram for ^99m^Tc-carbonyl core labeled gemifloxacin using 0.1% trifluoroacetic acid/water (Solvent A) and 0.1% trifluoroacetic acid/acetonitrile (Solvent B) as a mobile phase and the following gradient: 0 min 95% A (5% B), 5 min 95% A (5% B), 25 min 0% A (100% B), 30 min 0% A (100% B), flow = 1 mL/min, λ = 280 nm

**Figure 4:**
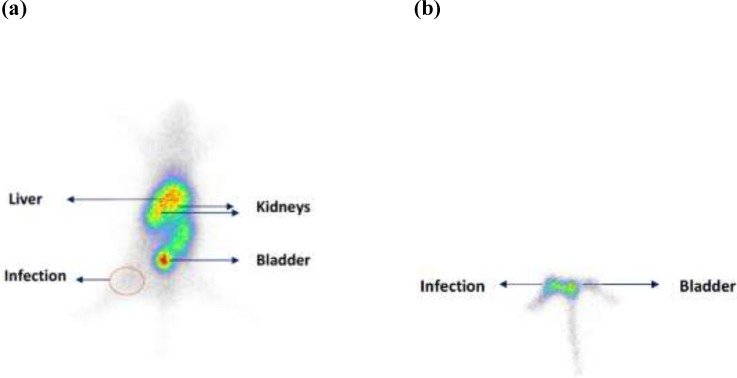
Scintigraphy of mice with right thigh muscle staphylococcus aureus infection 1 h post injection of ^99m^Tc(CO)_3-_Gemifloxacin. (a) hole body scan (b) Infection site (arrow show) after masking of abdominal region

**Table 1:** Influence of different amount of gemifloxacin ligand on carbonyl labeling efficiency. Values are expressed as mean ± standard deviation (n = 3

**Gemifloxacin (mg)**	**Labeling yield (%)**
0.5	3 ± 4.1
1.0	75.3 ± 3.6
1.5	90.4 ± 2.3
2.0	98.2 ± 1.1
2.5	97.8 ± 1.4
5.0	95.1 ± 2.1

**Table 2.** Biodistribution of ^99m^Tc(CO)_3-_Gemifloxacin in mice (%ID/g ± SD, n = 3

	**Post-injection time (h)**		
	**1**	**4**	**24**
Blood	12.15 ± 1.60	7.47 ± 1.35	2.57 ± 0.18
Heart	3.16 ± 1.12	3.23 ± 0.82	0.98 ± 0.15
Lung	8.51 ± 1.35	4.86 ± 0.82	1.72 ± 0.31
Stomach Bone	1.41 ± 0.51	1.53 ± 0.21	8.00 ± 1.39
Intestine	2.17 ± 0.64	2.11 ± 0.36	0.60 ± 0.29
Thyroid	5.74 ± 1.58	6.06 ± 0.72	1.37 ± 0.38
Liver	4.06 ± 1.33	3.34 ± 0.35	1.19 ± 0.21
Spleen	14.22 ± 1.38	13.18 ± 1.94	6.27 ± 0.95
Kidney	2.78 ± 0.35	1.83 ± 0.21	1.09 ± 0.28
Non-infected muscle	13.92 ± 0.47	8.59 ± 1.21	4.36 ± 0.75
*S. aureus *infected muscle	1.38 ± 0.21	0.89 ± 0.10	0.37 ± 0.04

Optimization of the radiolabeling condition was performed by varying reaction parameters such as the amount of ligand gemifloxacin and the pH value of reaction system, to identify an easy-to-use synthetic route giving a high radiolabeling yield of the product. Reducing the amount of ligand used in the formulation is still highly desirable. Therefore, one of the objectives was to reduce the amount of ligand gemifloxacin used in the labeling process. The amount of 2 mg gemifloxacin was the sufficient amount of ligand in formulation to reach a high labeling yield complex formation (Table 1). The effect of reaction pH was also investigated and the optimal pH range to produce a high labeling yield of ^99m^Tc(CO)_3_-gemifloxacin was found to be between pH = 4-5 and goes down to low labeling yield at pH out of this range (Figure 2).

Radiochemical purity of the ^99m^Tc-tricarbonyl core and ^99m^Tc-tricarbonyl-ligand were determined by TLC and reversed phase HPLC using the gradient systems consisted of 0.1% trifluoroacetic acid/water (Solvent A) and 0.1% trifluoroacetic acid/acetonitrile (Solvent B). The preparation yield for [^99m^Tc (CO)_3_(H_2_O)_3_]^+^ was > 95% acquired via TLC. In TLC the tricarbonyl precursors reveal a Rf of 0.3, and less than 3% of total activity was moved and counted in Rf of 0.7 belonged to ^99m^TcO_4_^-^ and only minimal activity (less than 1%) remained in origin corresponding to ^99m^Tc-colloid. The ^99m^Tc-tricarbonyl-gemifloxacin was characterized by HPLC which prepared in > 95% yield at specific activity of 0.14 GBq/μmol. The HPLC retention times of ^99m^TcO_4_^-^ was 4.19 min and for ^99m^Tc(CO)_3_-gemifloxacin was 15.53 min (Figure 3). 

The radiochemical purity of the ^99m^Tc-tricarbonyl-gemifloxacin was > 95% over the observed period of 24 h. No decomposition of the complex was observed in this time period, suggesting its high stability in normal saline at room temperature. Radiochemical stability of labeled antibiotic in human serum was 84 ± 2.4% after 24 h. So far the main drawback in labeling of ciprofloxacin and its similar structures are colloid impurity and instability which were the subjects of discussion in previous studies by different groups (16, 29-31) but here with design of a new labeling method by tricarbonyl core and optimization of labeling condition high labeling yield and stability for ^99m^Tc(CO)_3_-gemifloxacin is achieved.

The partition coefficient of the radiolabeled complex was determined by distribution in octanol and water, and the lipophilicity (log P) of ^99m^Tc(CO)_3_-gemifloxacin was found to be 0.7 ± 0.09, signifying its lipophilicity which could explain the accumulation of the radioligand in the liver*. In-vitro* binding of ^99m^Tc-tricarbonyl-gemifloxacin to S. aureus showed that 55 % of radioactivity bound to bacteria while no decomposition of ^99m^Tc labeled compound was observed in HPLC in evaluated condition. 

Biological evaluation of ^99m^Tc(CO)_3_-gemifloxacin complex was performed in Balb/c mice. The results are shown in Table 2. Clearance from the blood circulation was mild with 7.47 ± 1.35 %ID/g remaining in the blood at 4 h. Moderate clearance from the kidney (13.92 ± 0.47 %ID/g at 1 h and 8.59 ± 1.21 %ID/g at 4 h post injection) followed by slow liver clearance were observed (14.22 ± 1.38 %ID/g at 1 h and 13.18 ± 1.94 %ID/g at 4 h post injection). The presence of high activity in liver and kidney suggesting that the hepatobiliary and urinary systems are the major routes of excretion of the administered radioactivity.

The radioactivity concentration of infected muscle by S. aureus at 1 h post injection was 2.93 ± 0.30 %ID/g which decreased to 1.74 ± 0.01 %ID/g at 4 h post injection. The ratio of activity compared to non infected muscle was more than two fold (T/NT = 2.12 ± 0.24 at 1 h post injection) which was more than ratios reported previously for ^99m^Tc(CO)_3_-ofloxacin (1.63 ± 0.11 at 1 h post injection) (17). Results show that more than 90% the bounded activity in infected area at 1 h (T/NT = 2.12%) without any variation remains at 4 h period (T/NT = 1.95%) which is maybe due to the clearance of non specific uptake and on the other hand this high retention shows ^99m^Tc(CO)_3_-gemifloxacin have specific affinity to bacterial infection site, although the T/NT ratio at 4 h post injection is lower than 1 h post injection. 

Scintigraphic study at 1 h post injection showed uptake for radioligand in infections site (Figure 4). The uptake in all organs was decreased significantly after 24 h which shows that elimination is time depended and the early time up to 1 h is the best time for infection detection. 

## Conclusion

In this study, we have shown preparation and evaluation of an infection imaging agent ^99m^Tc(CO)_3_-gemifloxacin with high labeling yield. Based on the data obtained from this study, the product was stable, reproducible with high labeling efficiency with desirable characteristics making it a promising agent for imaging of infectious lesions. According to the results of *in-vivo* biodistribution studies, we found that this complex have infection site uptake with a good retention time. These promising characteristics make our new designed labeled conjugate as a very suitable candidate for diagnostic of infection sites in nuclear medicine. Further investigation on the ability to bind and detection of the other bacteria strains specially gram negative bacteria should be pursed.
